# Data on the decreased expression of FOXO1 by miR-1271 in HepG2 hepatocytes

**DOI:** 10.1016/j.dib.2017.10.055

**Published:** 2017-10-28

**Authors:** Won-Mo Yang, Kyung-Ho Min, Se-Whan Park, Wan Lee

**Affiliations:** a Department of Biochemistry, Dongguk University College of Medicine, Gyeongju-si, Gyeongsangbuk-do 38067, Republic of Korea; b Endocrine Channelopathy, Channelopathy Research Center, Dongguk University College of Medicine, Goyang-si, Gyeonggi-do 10326, Republic of Korea

**Keywords:** MicroRNAs, miR-1271, FOXO1, Hepatocyte

## Abstract

Obesity and metabolic diseases are closely associated with insulin resistance. Obesity-induced miRNAs are also considered to be potential contributors to the development of insulin resistance and type 2 diabetes. Previously, the expression of miR-1271 was reported to be upregulated in the liver of diet-induced obese mice (Yang et al., 2016) [1]. In this data article, multiple *in silico* analysis predicted FOXO1 gene to be a direct target of miR-1271. Dual luciferase reporter gene analysis showed that miR-1271 suppressed FOXO1 expression by direct binding to 3′UTR. The overexpression of miR-1271 reduced the protein expression of FOXO1, thereby reducing the transcription of PEPCK, a downstream target of FOXO1. The data is related to a research article entitled "MiR-1271 upregulated by saturated fatty acid palmitate provokes impaired insulin signaling by repressing INSR and IRS-1 expression in HepG2 cells" (Yang et al., 2016) [1].

**Specifications Table**

**Table:** 

Subject area	*Cell Biology*
More specific subject area	*Obesity, Metabolism, MicroRNA*
Type of data	*Figure and text*
How data was acquired	*Analysis of Dual luciferase reporter gene assay and immunoblotting*
Data format	*Analyzed*
Experimental factors	*Transfection of miR-1271, Analysis of FOXO1 and PEPCK expression*
Experimental features	*HepG2 hepatocytes were transfected with scRNA or miR-1271 mimic*. Expression of *FOXO1 was analyzed with Dual-luciferase reporter gene assay and immunoblotting. mRNA of PEPCK was determined by qRT-PCR.*
Data source location	*Dongguk University School of Medicine, Gyeongju-si, Gyeongsangbuk-do 38067, Korea*
Data accessibility	*The data are supplied with this article*

**Value of the data**

• The data are useful in understanding the regulatory relationship between miR-1271 and FOXO1 expression.
• The data can be compared with other obesity-related miRNAs involved in the pathogenesis of metabolic diseases.
• The modulation of miR-1271 expression can be applied further in functional studies of the cellular and systemic responses related with SFA-induced insulin resistance.

## 1 Data

**Fig. 1:**
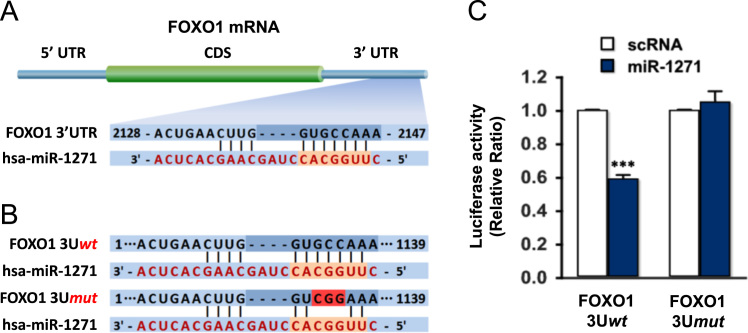
Targeting sites of miR-1271 in the 3′UTR of *FOXO1*, and an analysis of its binding by reporter gene assay. (A) The seed sequence of miR-1271 was predicted to target the 3′UTR of *FOXO1*. (B) For a Dual luciferase reporter gene assay, 3′UTR of the *FOXO1* gene was inserted downstream of a firefly luciferase open reading frame (wild-type; FOXO1 3U*wt*). The mutated 3′UTR of *FOXO1* gene lacking the miR-1271 binding sites was examined (mutant; FOXO1 3U*mut*). (C) Empty (lack of FOXO1 3′UTR), FOXO1 3U*wt* or FOXO1 3U*mut* pmirGLO vector was co-transfected with either scRNA control (open column) or miR-1271 mimic (closed column) into HepG2 cells. The relative luciferase activities were plotted against that of the FOXO1 3U*wt* cotransfected with the scRNA control, which was set to one. The values are expressed as the means ± SEM from at least three independent experiments. ***, P < 0.001 vs. scRNA control.

**Fig. 2:**
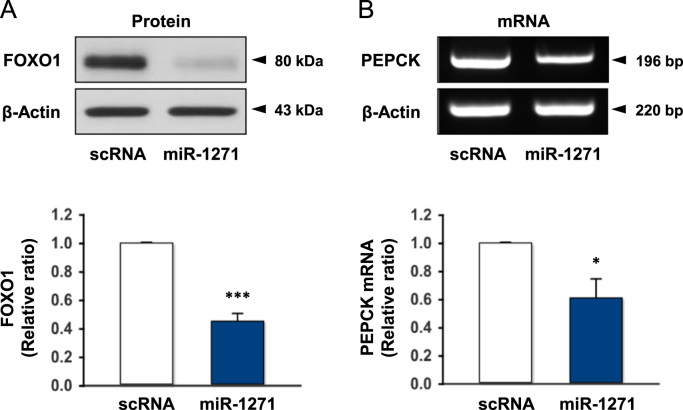
Effect of miR-1271 on the expression of FOXO1 and PEPCK. HepG2 cells were transfected with scRNA (200 nM) or miR-1271 (200 nM). (A) The protein expression of FOXO1 was analyzed after 48 h transfection by immunoblotting. The immunoblot is representative of four independent experiments. (B) The mRNA levels of PEPCK were analyzed at 48 h after reverse-transfection by RT-PCR (upper panel) and *q*RT-PCR (lower panel). The values are expressed as the relative ratio, where the intensity of the scRNA (open column) was set to one. Values are means ± SEM. *, P < 0.05; ***, P < 0.001 vs. scRNA control.

The expression of certain miRNAs targeting the molecules transducing insulin signaling is regulated aberrantly in saturated fatty acids (SFA)-induced obesity, and linked intimately to the pathogenesis of insulin resistance [2], [3]. Previously, a dysregulation of miR-1271 expression was reported to be linked causally to the development of hepatic insulin resistance [1]. This data article assessed the targets of miR-1271 on the insulin signaling pathway using *in silico* analysis, such as TargetScan, Pictar, and miRWalk. FOXO1 was found to be one such predicted target of miR-1271 that belongs to this pathway (Fig. 1A). This data article also presents accompanying data collected from a dual-luciferase reporter gene assay and immunoblotting to determine if FOXO1 would be a validated target of miR-1271 in hepatocytes. First, the direct binding of miR-1271 to *FOXO1* 3′UTR was determined using a Dual luciferase-based reporter assay. Luciferase reporter constructs containing either a tentative miR-1271 target sequence in *FOXO1* 3′UTR (wild-type; FOXO1 3U*wt*), or three nucleotides mutant of the tentative target sequence (FOXO1 3U*mut*) were generated in the pmirGLO vector (Fig. 1B), as described in the Section 2. These reporter constructs, which included a Firefly luciferase cassette to allow normalization of the internal Renilla luciferase activity, were transfected transiently together with a scRNA control or miR-1271 mimic. As shown in Fig. 1C, co-transfection with the miR-1271 mimic and reporter construct (FOXO1 3U*wt*) inhibited the luciferase activity compared to the scRNA control. Mutations in the tentative miR-1271 binding site in the *FOXO1* 3′UTR (FOXO1 3U*mut*) abrogated the repressive effect of miR-1271 (Fig. 1C). This suggests that miR-1271 targets *FOXO1* 3′UTR directly via its binding site. Moreover, the transfection of miR-1271 mimics decreased significantly the protein expression of FOXO1 in HepG2 cells, whereas the expression of the β-actin control was unaffected (Fig. 2A). Interestingly, the transfection of miR-1271 decreased significantly the mRNA level of PEPCK, a downstream target gene of FOXO1 (Fig. 2B). Further analysis of the data and insights into the implications of miR-1271 on the pathogenesis of insulin resistance and type 2 diabetes are reported elsewhere [1].

## 2 Experimental design, materials and methods

### 2.1 Cells and culture condition

HepG2, a human liver cancer cell line, was obtained from ATCC (#77400). The HepG2 cells were cultured in MEMα supplemented with 10% FBS and 1% penicillin-streptomycin (Gibco) in an atmosphere containing 5% CO_2_ at 37 °C. The cells from passages 4 to 10 were used for the subsequent experiments.

### 2.2 Plasmid constructs for Dual-luciferase reporter gene assay

The human *FOXO1* 3′UTR (1,200 nt) was amplified from HepG2 cells by RT-PCR with the forward primer: 5′- AAA AAG AGC TCC TTC ATT GGC TTG GTA TTT CC-3′ and reverse primer: 5′- AAA AAT CTA GAA TGC CAG GTT GGT CTG TTC G-3′. Human *FOXO1* 3′UTR, containing either the wild-type miR-1271 binding sites (FOXO1 3U*wt*) or the mutated miR-1271 binding sites (FOXO1 3U*mut*), was subcloned to the pmirGLO Dual-luciferase miRNA target expression vector (pmirGLO, Promega) with *Sac*I and *Xba*I. The target validation analysis was carried out using a Dual-luciferase reporter system (Promega) according to the manufacturer's instructions.

### 2.3 Transfection of miRNA mimics and plasmids

HepG2 cells were reverse-transfected with the scrambled control miRNA (scRNA) or miR-1271 mimics using G-fectin (Genolution, Seoul, Korea) according to the manufacturer's instructions. For the Dual-luciferase target validation assay, the HepG2 cells were co-transfected with scRNA or miR-1271 mimics and pmirGLO vector containing the reporter genes using Lipofectamine 2000 (Invitrogen).

### 2.4 RNA extraction and analysis of PEPCK mRNA

The total RNA from the HepG2 cells was extracted using a miRNeasy Mini Kit (Qiagen) according to the manufacturer's instructions. The purity and integrity of the RNA were assessed using a ND-1000 Spectrophotometer (NanoDrop) and Agilent 2100 Bioanalyzer (Agilent Technologies). A 2 μg of RNA from was reverse transcribed and the cDNA obtained was used to analyze the PEPCK transcript levels by RT-PCR and *q*RT-PCR with the forward primer: 5′-CAA TGC CGA CCT CCC CTG TG-3′ and reverse primer: 5′-CTG CTC CCG GTG TGG TGA TG-3′. RT-PCR and *q*RT-PCR were conducted using the GoTaq Green Master Mix (Promega) and SYBR Green PCR Master Mix (Applied Biosystems), respectively. The intensity data of *q*RT-PCR were analyzed by the advanced relative quantification method in Light-Cycler 480 software (Roche Diagnostics). β-Actin were applied as the internal control on the expression levels of the mRNAs.

### 2.5 Cell lysis, immunoblotting and antibodies

The cells were lysed using a lysis buffer and Laemmli solution. SDS-gel electrophoresis and immunoblotting analysis were performed, as described elsewhere [1]. The antibody against FOXO1 was purchased from Cell Signaling Technology. The proteins were visualized using an ECL Western Blotting Detection Reagent (GE Healthcare, Buckinghamshire, UK). The immunoblot intensities were quantified by densitometry using an analytical scanning system (Alpha Imager HP; Alpha Innotech, San Leandro, CA, US).

### 2.6 Database and statistical analysis

The miRNAs targeting the insulin signaling intermediates, such as FOXO1, were screened computationally with TargetScan, Pictar, and miRWalk analysis. The experimental values of all experimental are expressed as the means ± SEM from at least three independent experiments. Where applicable, the significance of the difference was analyzed using a Student's *t*-test for unpaired data.
